# Why primary obesity is a disease?

**DOI:** 10.1186/s12967-019-1919-y

**Published:** 2019-05-22

**Authors:** Antonino De Lorenzo, Santo Gratteri, Paola Gualtieri, Andrea Cammarano, Pierfrancesco Bertucci, Laura Di Renzo

**Affiliations:** 1 Section of Clinical Nutrition and Nutrigenomic, Department of Biomedicine and Prevention, University of Rome Tor Vergata, Rome, Italy; 2 Department of Surgery and Medical Science, Magna Græcia University, Germaneto, Catanzaro Italy; 3 Department of Laboratory Medicine, “Tor Vergata” University Hospital, Viale Oxford 81, 00133 Rome, Italy

**Keywords:** Obesity, Pathology, Phenotype, Obesity paradox

## Abstract

Obesity must be considered a real pathology. In the world wide, obesity represent one of the major public health issue associated with increased morbidity and mortality. Overweight or obesity, in fact, significantly increases the risk of contracting diseases, such as: arterial hypertension, dyslipidemia, type 2 diabetes mellitus, coronary heart disease, cerebral vasculopathy, gallbladder lithiasis, arthropathy, ovarian polycytosis, sleep apnea syndrome, and some neoplasms. Despite numerous informative campaigns, unfortunately, the fight against obesity does not seem to work: in the last years, the prevalence continued to increase. The progressive and rapid increase in the incidence of obesity, which has characterized most of the economically advanced countries in the last decade, has been the main stimulus for the research of the mechanisms underlying this pathology and the related disorders. The aims of this review is to provide a revision of the literature in order to define obesity as diseases, secondly to highlight the limits and the inaccuracy of common tools used for the diagnosis of obesity, and as a third thing to strengthen the concept of the complexity of obesity as a disease among political health care providers. Obesity may be viewed as a multifactorial pathology and chronic low-grade inflammatory disease. In fact, people affected by obesity have greater risk of developing comorbility and morbility, respect to healthy. Hence, the absolute therapeutic benefit is directly proportional to the basic risk. So, internationally interest on early diagnosis of obesity is growing to avoid under- and overdiagnosis consequences. Therefore, the consequences are an aggravation of the disease and an increase in obesity related pathology like diabetes, cardiovascular disease, and cancer. The most widely used parameter for diagnosis, body mass index (BMI) is not suitable for assessing the body fat. In fact, several studies demonstrate that BMI alone cannot define obesity, which consists not so much in weight gain as in excess fat mass. The use of suitable tools for the assessment of fat mass percentage combined with clinical and genetic analysis allowed to identify different phenotypes of obesity, which explain the various paradoxes of obesity. It is essential to adopt all possible strategies to be able to combat obesity, ameliorate the suffering of patients, and reduce the social and treatment costs of obesity.

## Background

World Health Organization (WHO) defined health as “a state of complete physical, mental and social well-being and not merely the absence of disease or infirmity”, with the fundamental aim to expand the conceptual framework of nations health systems. However, despite the efforts to improve or preserve health, chronic degenerative diseases have increased [1].

It therefore seems essential to define not only the state of health, but also of illness, in order to identify those indicators useful for promoting structural and environmental changes.

Diseases, that produce specific symptoms or affect a specific location, are defined as “deviations from the normal or healthy structure or function of a part, organ, or system of the body, caused by underlying etiologies, manifested by characteristic symptoms and signs, and resulting in pathologic consequences that affect health, feeling, or functioning” [2, 3].

The main requirement of defining the disease is its ability to accurately predict clinically relevant outcomes. It is important to underline that unclear definitions of illness result in inconsistent diagnoses and are of poor clinical utility.

In order to define a disease with certainty, it is necessary to know: the effects on incidence and prevalence of the disease itself; the changes to the natural history of the disease; the efficacy of the treatment; the benefits under conditions where the new definition will be used to determine the treatment threshold; the adverse effects including the psychological and economic ones; the usefulness of the definition of the disease on an individual and social level [4]. The possibility of not achieving all the listed requirements leads to a necessity of new definition [5].

The risk of an extension of the definitions of illness could be the inclusion of other pathologies, at different stages, already recognized.

In this case, both over- or underdiagnosis and a diagnostic error can led to no certainty of treatment efficacy. Due to this internationally interest on correct and early diagnosis is growing [6].

The critical point seems to lie in the lack of shared criteria for the definition of the disease and in the difficulty of the current ways of identifying and prevent any inappropriate changes [7].

However, from the point of view of a predictive and preventive medicine [8], the question that must be asked goes towards another direction. One has to wonder how the misdiagnosis or failure to early diagnosis of disease, such as obesity, aggravates the consequences in terms of morbidity and mortality and in health care costs, where correct diagnostic tools are available and personalized care is possible. A patient will benefit from the diagnosis of a disease only if it allows the understanding of symptoms or the risk of clinically relevant events, or if the patient can benefit from a specific treatment. In particular, the obesity problem seems to be the underdiagnosis rather than the overdiagnosis. In fact, about 30% of the people with obesity does not have a diagnosis because of the limit to evaluate the body fat content [9].

The aims of this review is to provide a revision of the literature in order to define obesity as diseases, secondly to highlight the limits and the inaccuracy of common tools used for the diagnosis of obesity, and as a third thing to strengthen the concept of the complexity of obesity as a disease among political health care providers.

## Obesity as disease and increased comorbidity risk

Obesity is a real epidemic and a public health problem, defined by The Obesity Society (TOS) as a disease [10], and not only an underpinning of major chronic diseases, but a serious debilitating condition in its own right [11].

Obesity is a multifactorial pathology that can be related to an altered nutritional behavior or secondary to genetic, hypothalamic, iatrogenic or endocrine diseases [12].

At the base of obesity is adiposopathy (or “sick fat”) defined as “pathologic adipose tissue anatomic/functional disturbances promoted by positive caloric balance in genetically and environmentally susceptible individuals that result in adverse endocrine and immune responses that may cause or worsen metabolic disease” [13].

Adiposopathy is sustained by adipocyte hypertrophy, visceral adiposity and ⁄or ectopic fat deposition and secretion of hormones, like leptin, and proinflammatory protein, like the plethora of cytokines, that in turn may lead to metabolic disease [14].

Therefore, we can classified obesity as a primary disease since the adiposopathy determines the dysregulation of the metabolic pathways [15]. Metabolic diseases most associated with primary obesity contribute to atherosclerosis, hypertension, dyslipidemia, diabetes type II, hyperandrogenemia in women and hypoandrogenemia/hyperestrogenemia in men [16].

There may be pathogenic immune and adiposopathic endocrine responses for the cardiovascular system or other systems. Sometimes, adiposopathy may cause atherosclerotic risk factors such as type 2 diabetes mellitus or dyslipidemia [17].

The diagnosis and treatment of obesity plays, therefore, an important role since this pathology is associated with an increased risk of numerous diseases and reduced life expectancy.

Pre-obesity or obesity significantly increases the risk of contracting and favoring the development of more than 200 chronic diseases [18], including but not limited to the following: type 2 diabetes mellitus (the risk of suffering from diabetes mellitus is 2.9 times higher in subjects with obesity, and the prevalence of diabetes is ten times greater in subjects with moderate obesity and thirty times in cases of excess of 135% of body weight compared to the ideal weight); cardiovascular diseases (CVD); hypertension (three-fold increase in risk of being affected by hypertension); dyslipidemia; coronary heart disease; gallbladder stones (three-fold increase in risk of being affected by gallbladder stones); obstructive sleep apnea syndrome; asthma; psychiatric diseases, including depression; polycystic ovary syndrome; nonalcoholic fatty liver disease; gastrointestinal reflux disease; osteoarthritis; some cancers. Among the most common tumors, obesity increases the risk of postmenopausal breast, endometrial, prostate, colorectal cancer and adenocarcinoma [19, 20]. Several studies on children have shown that there has been an increased risk of diabetes, CVD, cancer, PolyCystic Ovary Syndrome [21–25].

Obesity is necessary not only related to metabolic consequences and major chronic diseases, as it can considered a serious debilitating condition by itself. Excess of body fat may be accompanied by structural and functional abnormalities that reduced quality of life, as gastrointestinal reflux disease, gallbladder disease, osteoarthritis, obstructive sleep apnea/obesity hypoventilation syndrome, psychological and eating behavior disorders, anxiety and depression, and physical performance [10]. Moreover, obesity has an impact on cognitive functioning and major depressive disorder (MDD), with negative effects and additive on the processing speed and executive function measurements, as highlighted by mood rating questionnaires and neuropsychological tests [26].

Significant correlation among body composition variables, as weight, BMI, total body fat, and eating disorders, according to Eating Disorder Inventory-2 (EDI-2) score [27].

Furthermore, the excess of body fat reduces mobility, walking endurance and physical performance, accompanied by sarcopenia [28], regardless of age but according to the inflammatory status and genetic predisposition [29, 30].

The most serious consequences of obesity on health are hypertension, diabetes, myocardial infarction and major cardiovascular events. In particular, diabetes, a consequence of caloric excess, shows a direct association with other comorbidities, such as hypertension which is positively correlated due to vessel damage [31]. For this reason, the prevalence of cardiovascular complications has reached 64% only in older American patients who are obese and diabetic [32]. Between America, Europe and Australia the prevalence of hypertension reaches 60–75% [33–36]. In obese patients with diabetes and hypertension the incidence of ischemic heart disease exceeded 30% [37]. Heart failure is a serious condition and very widespread in obese patients with diabetes and hypertension, the prevalence in America, Europe and Austria reached 15%. In addition, the same category of patients presented with deep vein thrombosis and peripheral arteritis [38, 39].

In the obese, 30-day mortality after hospitalization for myocardial infarction reached 16% without diabetes [40] and 19% in those with diabetes [41]. Only in the US in 2012 there were over 110,000 deaths from cardiovascular disease [42].

Mortality due to comorbidities and to the increase in weight itself is a fact highlighted throughout the world in different populations [43]. This result suggests that preobesity and obesity alone are associated with increased mortality, thus bypassing the hypothesis that excess body fat in healthy subjects may play a metobolically protective role [44].

## Diagnostic methods and impact on prevalence of obesity

According to the classification based on body mass index (BMI), the ratio between weight in kilograms and height in meters squared (kg/m^2^) [45], a patient is considered overweight for BMI values greater than 25 kg/m^2^ and obesity is classified when BMI is greater than 30 kg/m^2^ [46].

For example, in USA, among adult men, the prevalence of obesity is: Hispanic, 37.9%; black, 38.0%; white, 34.7%; and Asian, 12.6%. In women, the prevalence of obesity is: Hispanic, 46.9%; black, 57.2%; white, 38.2%; and Asian, 12.4%. In children and adolescents, the prevalence of obesity is 17.0% of 2- to 19-year-olds subjects, with males and females equally affected [47]. The prevalence of obesity among US children and adolescents is: Hispanic, 21.9%; black, 19.5%; white, 14.7%; and Asian, 8.6% [48].

However, if we analyze the definition of obesity of the WHO, which identifies it as “a condition in which percentage body fat (PBF) is increased to an extent in which health and well-being are impaired”, we can state that it is defined by the expansion of the adipose tissue, rather than defining it solely on the basis of the increase in body weight [49]. The presence of obese subjects for BMI, or normal weight, with or without metabolic syndrome is a longstanding controversy. The research allowed to overcome this paradox with the introduction in the diagnosis of body composition, the evaluation of visceral fat, metabolic indices and genetic predisposition [50].

It is clear that the expansion of visceral and ectopic fat is a cardiovascular and metabolic risk factor that exceeds BMI. In clinical practice, measurements of visceral fat, adiposity, body composition and genetic/metabolic factors should be implemented to improve risk assessment and develop effective preventive and therapeutic strategies for high-risk obesity [51].

Therefore, BMI results limited and often unfit to discover hidden fat [52].

Obesity can be measured using direct and indirect measures of fatness other than BMI.

The body adiposity index has been found to be more sensitive to identify and classify obesity than the BMI [53]; however, it failed in the body fat estimation in populations with extreme amounts of fat [54].

Belarmino et al. validated a new anthropometric index to estimate the PBF, the Belarmino–Waitzberg (BeW) index, with a positive Pearson correlation (r = 0.74), a good accuracy (Cb = 0.94), and a positive Lin’s concordance correlation (CCC = 0.70) were observed comparing PBF estimated by air displacement plethysmography and BeW [55].

Anthropometric-based stratification is, however, prone to measurement errors. There are different limitations, such as age and ethnicities. Furthermore, these tools do not discriminate the types of visceral adiposity and the possible presence of metabolic risks.

In fact, as reported by Bray et al. and De Lorenzo et al. using BMI cutoff values to diagnose obesity approximately half of people with excess fat was missed, due to low sensitivity to identify adiposity [9, 47].

Therefore, it is necessary to use methods that accurately evaluate the amount of body fat (BF), fat free mass (FFM), skeletal muscle mass [56], the metabolically active body cell mass (BCM), bone mass, and the total amount of body water with the distribution of water compartments on large population samples [57].

The methods to estimate BF include, hydrostatic plethysmography, isotope dilution techniques, dual x-ray absorptiometry (DXA), skinfold method, bioelectrical impedance, magnetic resonance imaging, computed tomography scan and air displacement plethysmography [58–60].

Oliveros et al. from the Third National Health and Nutrition Examination Survey (NHANES III) tested the accuracy of BMI for diagnosing obesity in the adult general population using data from 13,601 individuals. Using bioimpedance analysis to calculate BF and BMI > 30 kg/m^2^ to define obesity, BMI had a very high specificity (97%) but poor sensitivity (42%) to detect obesity [52].

In a study of our group, we showed a wide range of PBF using dual energy x-ray absorptiometry (DXA) in people with normal BMI, ranging from 5.6 to 31.2% in men and from 4.6 to 51.1% among women. This underlines the main limitation of BMI, which cannot differentiate BF from lean mass, and central from peripheral fat [9].

De Lorenzo et al. comparing the classification of obesity according to the BMI with that according to the percentage of fat mass (25% for men and 30% for women), showed a strong discrepancy between the two measurements. In a study conducted in Italy on 900 subjects, of both sexes, aged between 18 and 83 years, it emerged that 28% of the participants classified as normal weight only on the basis of the BMI values resulted in pre-obesity condition, i.e. with a PBF above 25% in men and 35% in women, while 5% had obesity, with a PBF > 30% in males and > 40% in females. 50% of overweight subjects based on BMI values were in a condition of obesity [61]. Similarly, on other 3258 Italian subjects, the percentage of obesity changed depending on the criterion adopted. According to the BMI, obesity affected the 32.3% of population while, according to the *cut*-*off* acceptable percentage of fat mass as a function of sex and age, 64% of the population was in obesity status [9].

Therefore, a new predictive equation for PBF was evaluated, that could be helpful to clinicians to assess easily the body fat of their patients. PBF equation is available online at the site: http://www.mat.uniroma2.it/~ricerca/biosta/PBFcalculator.html [62].

The above results demonstrate that BMI alone is not able to define obesity, which consists not so much in weight gain as in excess of BF.

To overcome the limit of anthropometric assessment, due to heterogeneity of obesity, the edmonton obesity staging system (EOSS) was applied, as a tool useful for clinical staging system [63]. EOSS divides the population with excess adiposity on an ordinal 5-point scale, taking into account the comorbidities linked to obesity: (1) no apparent risk factors; (2) presence of obesity-related subclinical risk factors; (3) presence of established obesity-related chronic disease; (4) established end-organ damage; (5) severe disabilities. The possibility to predict mortality according to the EOSS was independent from BMI values. However, since EOSS cannot be used for a direct or indirect measure of adiposity, it represents only a prognostic system capable of integrating anthropometric indices [64].

To counter the increase in cases of obesity, the European Association for the Study of Obesity (EASO) has promoted various types of actions and has proposed the revision of the diagnostic criteria of the International Classification of Diseases ICD-11 [65]. In this context, the new definition of obesity as adiposity-based chronic disease (ABCD), for wich the term “adiposopaty” means the whole represented by the total quantity of fat, its distribution, and the function of adipose tissue, fits well with the EASO’s purposes [66]. Therefore, a diagnosis of ABCD could allow a more specific analysis of the complications caused by the dysfunctional adipose tissue, with a greater possibility of effectiveness of the intervention.

It appears that BMI classification should be overcome in favor of a new classification based on physiopathological findings. Therefore, as suggested by Penny Gordon-Larsen and Steven B. Heymsfield, the best strategy to prevent and treat obesity, recognized as disease and not behaviour, is to define the heterogeneity of obesity and its complication [67].

A WHO expert committee concluded “there is no agreement on the cut-off points for the PBF that constitutes obesity” [45].

Anyway, current research suggests that obesity cut-off points of PBF are in the 23–25% range in men and 30–33–35% range in women [68].

Furthermore, transversal and longitudinal studies have documented sex and age-related changes in body composition, confirming an age-related remodeling of body composition with decreased skeletal muscle mass, both in tone and in trophism, and a corresponding increase in visceral and intermuscular adipose tissue [69].

In order to avoid erroneous classifications, the diagnosis and treatment of obesity cannot be separated from a careful general and nutritional history, from the objective examination, from the measurement of the biochemical and hormonal parameters, from the measurement of energy expenditure to rest and, above all, the evaluation of body composition, in particular of the percentage of fat mass.

PBF turns out to be a better tool, compared to BMI, for a correct diagnosis of obesity, universally valid.

Advantages and disadvantages of diagnostic measures and index are resumed as follow:

### Limits of weight and height

In healthy adults, the fluctuations in weight are linked to daily physiological water, nutritional and evacuating changes of up to 2 kg, without representing loss or gain of lean or fat mass. In pathological states, imbalances may be greater. For example, in hemodialysis the weight can vary from 1.6 to 1.9 kg up to a gain of 4.0 kg between sessions [70]. Therefore, the measurement of vertical stature is prevented by confounding factors such as abnormal hair or curvature of the spine such as idiopathic scoliosis or muscular dystrophy [71]. Also, the use of height reported by the subjects should not be used in clinical practice, as demonstrated by several studies, especially in older women, affected by vertebral collapse, which tend to overestimate an average of 2.3–5 cm [72]. For these reasons, weight and height measurement must always be carried out at the same time of day associated with a clinical evaluation of the individual [73].

### Limits of circumference and skinfolds

The main limit of waist circumference and of waist/hip ratio is the impossibility of distinguish between subcutaneous and visceral adipose tissue at the abdominal level. This distinction is fundamental considering that visceral adipose tissue, compared to the subcutaneous tissue, is related to a high metabolic risk. The reference values (cut-off) are specific for country and population, but not specific for ages; this is a limit, as waist circumference generally increases in both men and women with their age. The reliability of the folds is limited by several factors, such as the variability of the thickness of the subcutaneous adipose tissue, the inter-individual variability of the elastic properties of tissues and the impossibility of measuring too large skinfolds reduce accuracy, especially in the obese. Furthermore, a limitation is the assumption that the amount of subcutaneous fat reflects that of visceral fat [74].

### Limits of the body mass index (BMI)

Although, BMI is significantly correlated with the amount of fat mass, measured with standard method, in the general population, the index loses predictability in the individual. Thus, individuals with the same BMI may have a significantly different fat mass [75]. The index, not including sex and age, tends to overestimate fat in young people and to underestimate it in the elderly. Since, the BMI does not evaluate individual body compartments. A value above the limits of normality is not always synonymous with an increase in fat mass: for example, an athlete could have a high BMI but a reduced fat mass and still be defined as overweight or obese [76].

### Advantages and disadvantages of bioimpedentiometry analysis (BIA)

The BIA is characterized by simplicity of use, repeatability, low cost and invasiveness. Furthermore, this is recognized as a standard for the evaluation of the body cell mass. On the contrary, potential errors of impedance analysis are mainly due to an altered state of hydration and/or electrolyte balance that interfere with tissue impedance. Attention to pre-test, fasting and rest protocols is essential to ensure that the measurement obtained is as accurate as possible [77]. The use of BIA for the body composition in clinical practice is less accurate in the obese, nephrological and altered hydration patient. It is also important to remember that BIA was developed for the analysis of the body composition of healthy adults with normal and constant hydration. However, it must be pointed out that BIA can specifically recognize and measure only the conductive compartment of tissues. Moreover, the conductivity of the body’s tissues is determined not only by hydration, but also by the limits imposed by the individual’s body geometry, according to its phenotype. Body geometry is a key point. The overweight/obese phenotype, with mainly android fat distribution, moves away from the five-cylinder ideal model, and is more susceptible to errors in the estimation of body composition [78].

### Advantages and disadvantages of dual-energy X-ray absorptiometry (DXA) analysis

The DXA analysis is the standard for the tri-compartmental body composition, fat mass, lean and bone. In addition, densitometric analysis allows evaluation on both whole and segmental bodies. The newly released software is able to extrapolate the visceral fat mass thus applying a body composition method to the evaluation of cardiovascular risk. The technical limits of DXA are the maximum weight limit (200 kg) and the restrictions for height and wide of the individual examined (197 × 66 cm), compromise the accuracy of the measurement with DXA for subjects outside normality range. Moreover, specific algorithms are necessary for the estimation of fat mass hidden by the bone shadow cone. DXA does not provide an estimate of the hydration status, since it measures neither total water nor the hydration of lean mass. However, it remains to take into account inter-individual variability related to food and fluid intake, physical exercise and other physiological or pathological processes, which alter body water content [79].

## The “obesity paradox” (OP) and the “obesity phenotypes”

The possibility of using increasingly precise and accurate biomarkers, useful for a precise diagnosis and a personalization of therapy, and the identification of paradoxes would seem to justify the doubts that lead to define overdiagnosis as the worst event of underestimation of the disease.

However, if we try to analyze the various paradoxes, we will see how often these may depend on the wrong methods used for the diagnosis itself.

Analyzing the risk factors and illness scoring systems that included BMI in their risk adjustment, as the Acute Physiology and Chronic Health Evaluation (APACHE) IV system [80], the Oxford Acute Severity of Illness Score (OASIS) [81], the Sequential Organ Failure Assessment (SOFA) score [82], and the Simplified Acute Physiology Score (SAPS) 3 [83], a counterintuitive epidemiological phenomenon is observed, defined as “re-verse epidemiology” or “obesity paradox.” [84]. In the case which the diagnosis of malnutrition, due to excess of body fat mass and/or a defect of body lean mass, does not take into account the equilibrium and cross-talk between fat and muscle mass, patients with morbid or severe obesity tend to have lower morbility, and hospital mortality rates than patients with normal weight or underweight [85]. Potential confounders may be relevant to explain this paradox. According to Deliberato et al. this depends on the fact that weight itself, and therefore the BMI, might not be explain the extreme obesity [86].

The “obesity paradox” (OP) could be explained by the fact that the classification with BMI obesity definition groups together, in the same category, subjects with different clinical and biochemical characteristics. In multiple investigations, obesity measured by BMI and various other indices has been linked to the survival of heart failure (HF) [87].

In a large population of 7499 individuals with symptomatic HF, with preserved and reduced ejection fraction, there is an improvement in the quality of life and a reduction in the risk of mortality in subjects with obesity. The group with the highest BMI (35 kg/m^2^) had similar risk to those with BMI 30.0–34.9 kg/m^2^ [88].

Moreover, lower BMI was associated with a greater risk of all death causes in patients without edema, because edema increased BMI based on fluid excess rather than solid tissue mass [89].

The BMI classifies in the same categories individuals with different clinical and biochemical characteristics without taking into account inflammatory status of visceral adipose tissue, related to the risk of CVD [90]. Hypertrophic or apoptotic adipocytes in individuals with obesity increased the pro-inflammatory status, due to the secretion of several molecules such as leptin, resistin, interleukin 6 (IL-6) and tumor necrosis factor-α (TNF-α) that can activate M1 macro-phage-response [91, 92]. Therefore, Vecchiè et al. highlighted the need for an approach that consider the heterogeneity of obesity [93]. This is explained by the fact that BMI is not a parameter that can be used for diagnosis, nor for fat distribution, or based on age and gender. In fact, the amount of adipose tissue and distribution of adiposity were significantly related to adverse cardiac remodeling and adverse hemodynamics [94].

The OP does not only occur in CVD patients, but also exists in other chronic diseases.

A potential protective effect of high BMI in terms of improvement in kidney disease and end-stage renal disease was evaluated. With a glomerular filtration rate < 60 ml/min/1.73 m^2^ or presence of microalbuminuria, a BMI of 18.5–22 kg/m^2^ was associated with a higher risk of death [95].

Kalantar-Zadeh et al. demonstrated that survival depends on muscle mass. Long term, muscle gain with a loss of total body weight is better than weight gain with loss of muscle mass [96]. Patients with obesity are less inclined to develop cachexia, and protein-energy wasting. Moreover, less intradialytic hypotension was observed [95].

Many papers confirmed the existence of an OP in type 2 diabetes mellitus [97], underling the U-shaped association between mortality and BMI [98, 99].

All this does not mean that patients with chronic degenerative diseases have to weigh down [100]. But, it is rather important to evaluate body composition, the person’s weight history, the type of previous or in course medication and behavioral therapy (i.e., diet and physical activity changes). All this because it is possible that higher mortality in normal weight subjects may be associated with low muscle mass and not low adiposity [101].

The existence of many “obesity phenotypes” with different metabolic and CVD associated risk due to physical and life-style features, underlining the heterogeneity of obesity, which recognizes numerous possible etiologies, could explain in part the described paradoxes [76, 93].

De Lorenzo et al. [59] classified different phenotypes of obesity: the normal-weight obesity (NWO); the metabolically healthy obesity (MHO); the metabolically unhealthy obesity (MUO).

The metabolic disorders were evaluated according to National Cholesterol Education Program Adult Treatment Panel III (NCEP ATP III) to distinguish MHO from MUO, and according the International Diabetes Federation (IDF) criteria for the differential diagnosis between NWO syndrome and MONW [102].

Furthermore, an additional phenotype of ‘super-obesity’, that designate patients with a BMI ≥ 50 associated with a greater burden of obesity-related comorbidities, representing about 5–10% of all obese persons, was highlighted [103]. Antonini-Canterin et al. demonstrated that superobesity was associated with insulin resistance and a worse impact on cardiac remodeling and left ventricular diastolic function [104].

Metabolically healthy subjects also exist in the super obese phenotype. In particular, it has been shown that men suffering from superobesity find themselves in a better metabolic condition than women; the beneficial metabolic situation could be explained by sex hormone changes that favor gynoid fat distribution [105].

In particular, two sub-classes of NWO were described [52]: the metabolically healthy normal weight obesity, typical of Normal Weight Obese (NWO) syndrome [91], with high CVD risk indices, and the normal weight obesity associated with Metabolic Syndrome (MetS) and insulin resistance [106] defined as metabolically obese normal weight (MONW) [107].

Therefore, to identify subjects with cardiometabolic risk, it is crucial to evaluate not only the BMI but also the total amount of BF, the regional distribution of fat and ectopic fat depots, due to the secretion of different adipokines and other bioactive substances that are related to metabolic disorders [108].

MHO individuals show a BMI > 30, a PBF > 30%, and waist circumference > 90 cm, with normal lipid and blood pressure profile, and a good insulin sensitivity [109]. In MHOs, the higher levels of insulin sensitivity and inflammation may be due in part to a reduction in visceral adiposity compared to a large amount of total fat [110]. Blood pressure is normal, serum lipid profile is well preserved, the degree of inflammation is low and no abnormal liver function is observed; MHO individuals are usually young, with good levels of physical activity and a good dietary habit [111].

Other metabolic, genetic or behavioral factors could be involved and should be investigated with appropriate studies [112]. Furthermore, many doubts remain regarding the understanding of the factors contributing to the protective profile of these individuals [113]. MHO and subclinical atherosclerosis are mediated by metabolic risk factors, which are well below as effectively considered as abnormal in routine lab tests [114].

It follows that these individuals cannot be considered in an optimal state, but rather that they present a lower risk than the subjects with obesity “at risk”, but still greater than in the general population.

NWO and MONW subjects are mostly unaware that they are in the risk group, and MHO individuals need medical attention and periodic weight management because of obesity-related complications [115–117].

MONW are subjects with weight and BMI in the normal range, but with high PBF and a cluster of abnormalities related to obesity. These individuals are usually young and show premature signs of insulin resistance, hyperinsulinemia, dyslipidemia that could be associated with an increased risk of diabetes and CVD [107].

In 2006, our group for the first time described the NWO syndrome with metabolic abnormalities. This syndrome is characterized by normal weight individuals but genetically obesity, in an early low-grade inflammatory state [118].

The PBF is higher than 30%, with is a significant reduction of the lean mass, equal to at least 1.5 kg (FFM kg), particularly in the lower limbs muscle mass [30]. The effects of the body composition on the Resting Metabolic Rate, assessed by indirect calorimetry, result in a reduction of about 200 kcal per day, which can be explained by a reduction of the metabolically active lean mass. NWO individuals exhibit a narrow inverse relationship between cardiovascular risk indices and body fat distribution [119, 120].

The unified theory explained the relationship between inflammation and chronic diseases, but it is essential evaluate how genetic characteristics interact with the exposition to environmental agents, defining the phenotype [121].

They have high circulating levels of TNF-α, interleukins as IL-1α, IL-1β and IL-8 [122], and an oxidative stress related to metabolic abnormalities [123].

NWO show an alteration of a cluster of genes linked to inflammation and aging. NWO women have specific and characteristic polymorphisms of IL-15 receptor alpha subunit and 677 C/T methylenetetrahydrofolate reductase (MTHFR) genes, which increase the risk of breast cancer, carcinoma of the colon and sarcopenia [119].

A polymorphism in the second intron of the IL-1 receptor antagonist gene is associated with NWO syndrome, predisposing to ovarian, pancreatic, cervical and gastric carcinoma risk [123].

The -308 G/A TNF-α polymorphisms increased the prevalence of sarcopenia in NWO: considering appendicular skeletal muscle mass index values, 4.21% of NWO subjects were sarcopenic [30].

Investigating the allelic frequency of the TP53 codon 72 in exon 4 polymorphism, the risk of being sarcopenic for *Arg/*Arg genotype in NWO women is 31% higher than normal weight lean carriers [29].

In the United States of America the NWO was present in ~ 30 million Americans, with heightened cardiometabolic risk [80]. In Switzerland the frequency of NWO was 10.1% in women and 3.2% in men [124].

Marques-Vidal et al. [125] found that NWO women had higher blood pressure and greater prevalence of dyslipidemia and hyperglycemia than lean women did [126].

Evaluating the cardiovascular mortality risks, Sahakyan et al. demonstrated that women with NOW, with visceral adiposity had a higher mortality risk than those with similar BMI but no visceral obesity (HR, 1:48 [CI 1:35 to 1.62]) and those who were in obesity status according to BMI only (HR, 1:32 [CI 1.15 to 1.51]) [127].

Kang et al. highlighted for the first time that NWO subjects have a higher degree of vascular inflammation using 18 F-fluorodeoxyglucose positron emission tomography/computed tomography (18 F-FDG-PET/CT) respect to normal weight lean subjects [128].

In 23.748 Chinese people the prevalence of NWO was 9.5% for men, 6.6% for women, associated with increased cardiometabolic risks and MetS, also after excluding the effect of abdominal obesity [129]. Liu et al. showed that waist-to-height ratio (WHtR) and waist-to-hip ratio (WHR) are the optimal indicators of MetS in Chinese postmenopausal women [130]. Chinese NWO postmenopausal women in the highest WHtR tertile (≥ 0.05) had a higher odds ratio for the presence of at least 2 non-adipose MetS components (p < 0.05) compared with NW women in the lowest tertile after adjusting for confounding factors, such as age and metabolic parameters [131]. This is the result of the clinical assessment of WHtR, which should be conducted to prevent adverse CVD risk in postmenopausal women, independently of BMI.

## Conclusions

Overall, these results increasingly support the importance of defining the precursory indicators of obesity phenotypes with a view to identifying possible morbidity and mortality risk indexes, in order to establish targeted diet therapy as soon as possible, also considering possible variants genotypes found [132].

It is essential to adopt all possible strategies to be able to combat obesity, ameliorate the suffering of patients, and reduce the social and treatment costs of obesity.

Therefore, an early identification of all obesity phenotypes is fundamental, as they constitute a “vulnerable” category because, based on the indices and measures adopted to classify obesity, they are not aware of being at risk of developing pathologies linked to obesity.

Cost of illness (COI) assist policy to recognize the financial load of a disease. Obesity leads to a big expenditure on medicine care, private and public costs. It is a priority defines actions of prevention in order to save on social resources public [133].

Since obesity is a complex disease condition with much different co-morbidity, what fraction of the co-morbidities is attributed to obesity has much influence on the cost calculation. If we assume that at least 10% of the adult over 18-year-old population with normal weight is actually in obesity status according the PBF, the COI of obesity would increase and the 5–6% spend more than if they are not taken care [134, 135].

Treatments of severe obesity resulted in high degree of heterogeneity. In particular, Ryder et al. demonstrated that lifestyle, pharmacotherapy, or metabolic and bariatric surgery interventions ranged from − 50.2 to + 12.9% of BMI reduction; therefore, it is necessary to identify new precision medicine approaches to counter obesity [136, 137].

Biochemical and body composition indices, associated with genetic analysis and the study of the inflammatory pattern, are fundamental clinical tools for the diagnosis of obesity but also to predict, with years of advance, the development of type II diabetes, cardiovascular disease and cancer [131, 138].

The fallout of a correct and early diagnosis of obesity will also produce lower health costs for primary and secondary prevention of the most common degenerative diseases related to it.

## Data Availability

Not applicable.

## Abbreviations

WHO: World Health Organization
TOS: The Obesity Society
CVD: cardiovascular diseases
BMI: body mass
PBF: percentage body fat
BeW: Belarmino–Waitzberg
BF: body fat
FFM: fat free mass
BCM: body cell mass
DXA: dual X-ray absorptiometry
OP: obesity paradox
HF: hearth failure
IL: interleukin
TNF-α: tumor necrosis factor-α
NW: normal weight
NWO: normal-weight obese
MHO: metabolically healthy obese
MUO: metabolically unhealthy obese
MetS: metabolic syndrome
MONW: metabolically obese normal weight
WHtR: waist-to-height ratio
WHR: waist-to-hip ratio
COI: cost of illness
